# Early Experience of TACE Combined with Atezolizumab plus Bevacizumab for Patients with Intermediate-Stage Hepatocellular Carcinoma beyond Up-to-Seven Criteria: A Multicenter, Single-Arm Study

**DOI:** 10.1155/2023/6353047

**Published:** 2023-04-15

**Authors:** Kang Wang, Hongfei Zhu, Hongming Yu, Yuqiang Cheng, Yanjun Xiang, Zhangjun Cheng, Yang Li, Tao Li, Dongxu Wang, Zhenyu Zhu, Shuqun Cheng

**Affiliations:** ^1^Department of Hepatic Surgery VI, Eastern Hepatobiliary Surgery Hospital, Second Military Medical University, Shanghai, China; ^2^Hepato-Pancreato-Biliary Center, Zhongda Hospital, School of Medicine, Southeast University, Nanjing, China; ^3^Department of Liver Surgery, The Third Affiliated Hospital of Sun Yat-sen University, Guangzhou, China; ^4^Department of Hepatobiliary Surgery, Qilu Hospital of Shandong University, Jinan, China; ^5^Department of Hepatobiliary Surgery, The Fifth Medical Center of Chinese PLA General Hospital, Beijing, China

## Abstract

**Aim:**

Locoregional treatment, such as TACE, in combination with immunotherapy may elicit a synergistic anticancer effect. However, TACE combined with atezolizumab plus bevacizumab (atezo/bev) has not been investigated for patients with intermediate stage (BCLC B) HCC beyond the up-to-seven criteria. This study aims to evaluate the efficacy and safety of this treatment strategy in intermediate-stage HCC patients with large or multinodular tumors exceeding the up-to-seven criteria.

**Methods:**

This multicenter retrospective study included patients with intermediate stage (BCLC B) HCC beyond the up-to-seven criteria treated with TACE combined with atezo/bev from five centers in China from March to September 2021. The outcomes of this study included the objective response rate (ORR), overall survival (OS), and progression-free survival (PFS). Treatment-related adverse events (TRAEs) were analyzed to assess safety.

**Results:**

A total of 21 patients were enrolled in this study, with a median follow-up duration of 11.7 months. According to Response Evaluation Criteria in Solid Tumors (RECIST) version 1.1, the best ORR was 42.9% and the DCR was 100%. According to modified RECIST (mRECIST), the best ORR and DCR were 61.9% and 100%, respectively. The median PFS and OS were not reached. The most common TRAEs at all levels were fever (71.4%), and the most common grade 3/4 TRAE was hypertension (14.3%).

**Conclusions:**

TACE combined with atezo/bev showed encouraging efficacy and an acceptable safety profile, making it a promising treatment option for patients with BCLC B HCC beyond the up-to-seven criteria, which will be further investigated in a prospective single-arm trial.

## 1. Introduction

Hepatocellular carcinoma (HCC) is the sixth most common cancer and the fourth leading cause of cancer-related deaths worldwide [1]. The Barcelona Clinic Liver Cancer (BCLC) algorithm, which was introduced in 1999, measured both tumor burden and liver function and is the most commonly used staging system worldwide [2, 3]. Patients with intermediate stage HCC defined, as BCLC stage B constitute a large subgroup accounting for 20–30% of cases [4, 5]. The prognosis of these patients is unsatisfactory, especially for patients with BCLC B HCC beyond up-to-seven criteria [6, 7]. Treatment of patients with intermediate stage HCC remains challenging.

Transarterial chemoembolization (TACE), as the recommended treatment for intermediate stage patients, has been used more than 30 years [8]. However, not all patients benefit from TACE because intermediate-stage HCC is a very heterogeneous disease in terms of tumor burden and liver function [9, 10]. The latest BCLC version released in 2022 stratifies the BCLC-B into three groups of patients and systemic treatment is also recommended, especially for patients with diffuse, infiltrative, or extensive bilobar liver involvement [6]. Systemic treatments with molecular and immune therapies have dramatically changed the management of HCC. The IMbrave 150 trail demonstrated superior results for atezolizumab plus bevacizumab (atezo/bev) to sorafenib for patients with unresectable hepatocellular carcinoma, which heralded a new era of combination treatment [11]. The objective response rate (ORR) for patients with intermediate stage HCC receiving the treatment of atezo/bev is 44% [11]. Furthermore, the ORR of atezo/bev in patients with HCC beyond the up-to-7 criteria is only 17.7% per the response evaluation criteria in solid tumors (RECIST) criteria at six weeks, which is not satisfactory [12]. Therefore, new treatment strategies are needed to improve therapeutic efficacy.

There is increasing interest in the combination of immunotherapy and locoregional therapies for the treatment of HCC. A number of clinical trials have been initiated to assess the efficacy of combination with molecular and immune therapies plus locoregional therapies [13, 14]. The rationale for this combination strategy is sound. Local therapies, such as TACE, could lead to the release tumoral neoantigens and proinflammatory cytokines, whereas vascular endothelial growth factor (VEGF) inhibitors could enhance immunity and prime tumors for checkpoint inhibitors [14, 15]. Previous studies have shown the promising results in the treatment of advanced HCC with the triple combination of tyrosine kinase inhibitors, immune checkpoint inhibitors, and locoregional therapy [16–18]. However, the efficacy of atezo/bev plus TACE has not been reported in the treatment of intermediate stage of HCC.

Hence, this multicenter retrospective study enrolled BCLC-B patients with HCC beyond the up-to-seven criteria who accepted atezo/bev and TACE and investigated the efficacy and safety of the combined therapy.

## 2. Methods

### 2.1. Patient Selection

Patients with intermediate stage HCC beyond the up-to-seven criteria who were treated with atezo/bev and TACE as first-line therapy at Eastern Hepatobiliary Hospital, Qilu Hospital, The Third Affiliated Hospital of Sun Yat-sen University, Zhongda Hospital, and The Fifth Medical Center of PLA General Hospital from March to September 2021 were enrolled in this retrospective study. Eligible patients were aged 18 years or older, diagnosed as HCC staged at BCLC B, treated with atezolizumab plus bevacizumab in combination with TACE, adequate liver function; an Eastern Cooperative Oncology Group performance status (ECOG PS) of 0-1, at least one measurable target lesion as per the Response Evaluation Criteria in Solid Tumors (RECIST) criteria (version 1.1) and adequate hematologic and organ function. Exclusion criteria included prior treatment, a history of autoimmune disease, and untreated treated esophageal or gastric varices.

This study was in compliance with the Ethical Standards of Declaration of Helsinki, and was approved by the Institutional Ethics Committee of each center. All patients provided written informed consent and agreed to provide their archive and tumor tissue before conducting the treatment.

### 2.2. Diagnosis and Treatment

HCC was diagnosed based on alpha-fetoprotein (AFP) levels, dynamic computed tomography, dynamic magnetic resonance imaging, or pathology findings [19, 20]. Conventional TACE was recommended as prior treatment for patients who were diagnosed with BCLC B stage HCC. In addition, atezolizumab combined with bevacizumab was also recommended because of their promising antitumor potentiality and the final decision was principally up to the patient. TACE was initiated before the first cycle of atezo/bev. Sequential TACE was performed on demand while treatment with atezo/bev was continued.

TACE was conducted using a Seldinger technique with femoral arterial puncture under local anaesthesia. Arterioportograms were performed to assess tumor staining and vascularity. The hepatic artery supplying the tumor was cannulated selectively. After catheterization of the arteries, doxorubicin hydrochloride, pirarubicin or pharmorubicin, and lipiodol were injected through the catheter. The amounts of lipiodol and doxorubicin were adjusted according to the body surface area of the patient and liver function. Gelfoam fragments were then injected to embolize the tumor feeding vessels until stasis of blood flow was achieved. TACE could be divided into two treatment sessions in the case of multifocal disease if deemed appropriate by the interventional radiologist. Repeated TACE is performed when there is evidence of residual tumor, with adequate liver function and the absence of contraindications such as portal vein trunk thrombosis, uncontrolled infection, or comorbidities that would preclude the procedure.

A fixed-dose of 1,200 mg of atezolizumab injection will be intravenously administered on Day 1 and Day 22 (60 min of initial IV infusion, followed by 30 min if tolerated), and bevacizumab 15 mg/kg will be intravenously administered after an interval of at least 5 min (90 min of initial IV infusion, followed by 60 min and 30 min if tolerated). In case of severe toxicity, dose adjustments for atezolizumab and bevacizumab were performed according to the drug's instructions.

### 2.3. Follow-Up and Outcomes

All patients were followed up every 6–8 weeks. At each follow-up visit, there was a routine history of physical examination, laboratory blood tests, and an enhanced CT/MRI. Assessment of tumor progression was based on RECIST 1.1 and mRECIST. The most recent follow-up visit was on May 20, 2022.

The primary outcome of this study was the objective response rate (ORR), defined as the proportion of patients with a complete response (CR) or partial response (PR) per the investigator's assessment. The secondary outcomes included overall survival (OS) and progression-free survival (PFS). OS was defined as the time from the commencement of TACE to death from any cause or the date of the most recent follow-up. PFS was defined as the time from the commencement of TACE to progression, death from any cause, or the most recent follow-up. We additionally calculated durable response rate (DRR) ≥ 3/6 months (continuous complete or partial objective response lasting ≥3/6 months) to assess treatment efficacy [21].

Adverse events were assessed according to the National Cancer Institute Common Terminology Criteria for Adverse Events (CTCAE) version 5. Clinical and radiological data for diagnosis were collected from the case record.

### 2.4. Statistical Analysis

Kaplan–Meier analysis was used to illustrate the PFS and OS. Quantitative data were expressed as the means and range, and categorical data were expressed as a number (percentage). IBM SPSS Statistics 22 and R 4.0.2 software (https://www.r-project.org/) were used for data analyses.

## 3. Results

### 3.1. Patients

Twenty-three patients with intermediate HCC beyond up-to-seven criteria received the treatment of atezo/bev in combination with TACE in 5 different institutions between March 1, 2021, and August 30, 2021. Two patients failed to perform the image tests and were subsequently lost to follow up. Thus 21 consecutive patients were enrolled in the analysis. At the data cutoff (May 30, 2021), the median follow-up was 11.7 (range, 7.5–15.0) months.

The baseline patient characteristics are summarized in Table 1. Median age was 56 years and nineteen patients were male. 20 (95.2%) patients had Child-Pugh grade A liver function and 1 (4.8%) patient had Child-Pugh grade B7. When using modified albumin-bilirubin (mALBI) grade to assess hepatic function, 19 (90.5%) patients were grade 1/2a. The median cycle of atezo/bev use was 9 (range, 1–20) and patients received the first cycle of atezo/bev 5–10 days later after TACE was done. The median number of TACE procedures was 2 (range, 1–4). Six of the twenty-one patients received one session of TACE while twelve patients received two sessions of TACE during the treatment period.

### 3.2. Efficacy

Tumor response was evaluated based on the investigator's assessment per RECIST v1.1 and mRECIST. The best tumor response is shown in Table 2. According to RECIST version 1.1, the ORR was 42.9% and DCR was 100%. While ORR was 61.9% and the DCR was 100% according to mRECIST.

**Figure 1:**
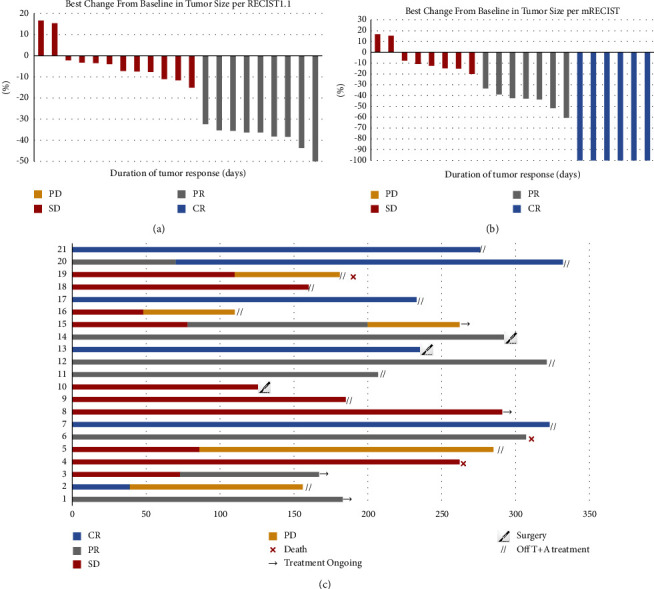
Characteristics of objective response in patients with TACE combined with atezolizumab plus bevacizumab. (a) The maximum percentage reduction from baseline in primary tumor per RECIST1.1. (b) The maximum percentage reduction from baseline in primary tumor per mRECIST. (c) Duration of response.

**Figure 2:**
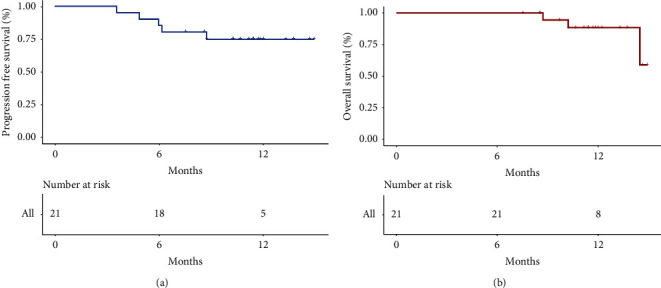
Kaplan–Meier curves for progression-free survival (a) and overall survival (b).

**Figure 3:**
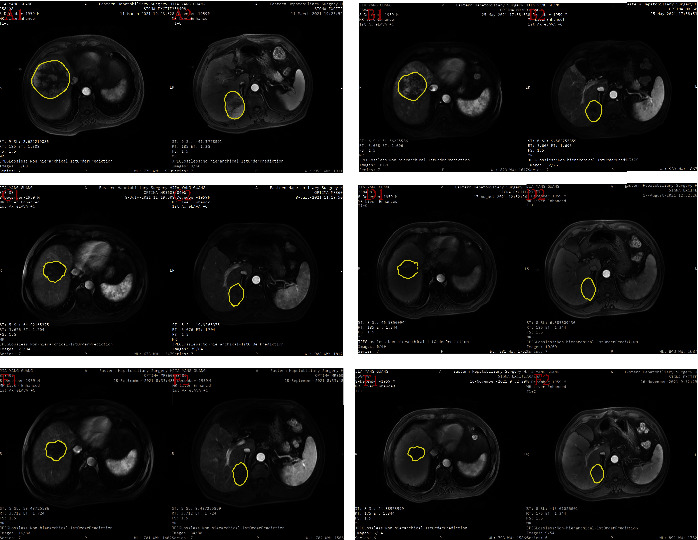
Response to combined TACE and atezo/bev therapy. Longitudinal imaging in a 61-year-old with history of hepatitis B viral infection. The patient was diagnosed with unresectable HCC on March 11, 2021 and then began triple combination therapy. First TACE was performed on March 16, 2021, and then atezo/bev was initiated on March 18, 2021 and repeated every 3-4 weeks. The second TACE was performed on May 26, 2021 (A1, A2). Imaging manifestations of the patient before the treatment, showing two lesions in the right lobe of the liver (B1, B2). Imaging manifestations of the patient after 1 TACE session and 4 cycles of atezo/bev, the efficacy evaluation showed PR per mRECIST (C1, C2). Imaging manifestations of the patient after 2 TACE sessions and 6 cycles of atezo/bev, showing that the lesions in the right lobe of the liver were generally necrotic. The efficacy evaluation showed CR per mRECIST (D1–F2). Imaging manifestations of the patient after 11 cycles of atezo/bev, showing that the lesion in segment 8 was significant shrinkage and lesion in segment 6 remained no enhancement.

The changes from baseline in target lesions according to RECIST 1.1 and mRECIST are shown in Figures 1(a) and 1(b). The description of the following results is based on mRECIST by default. Patient details of response durations and outcomes are presented in Figure 1(c). At the last follow-up, 3 patients died: (1 from tumor progression and 2 from liver failure). Five patients are continuing treatment with atezo/bev, eleven patients discontinued treatment (four due to tumor progression, two due to stable tumor and five due to tumor complete response or partial response), and three patients underwent hepatectomy. The median OS was not reached with a 1-year OS rate of 90.5%. The median FPS was also not reached with a 1-year PFS rate of 76.2% (Figure 2). DRR ≥3 months was 57.1% and DRR ≥6 months was 47.6%. Five patients underwent PD. First, they will be recommended other first-line treatment options, such as lenvatinib or sorafenib. A patient was evaluated as CR per mRECIST after treating with TACE combined with atezo/bev, as shown in Figure 3.

### 3.3. Safety

Treatment-related adverse events were evaluated based on frequency and severity according to CTCAE version 5.0. Each patient experienced at least one adverse event in the duration of treatment (Table 3).

The most common TARE included fever (71.4%), aspartate transaminase elevation (66.7%), alanine aminotransferase elevation (61.9%), fatigue (47.6%), and abdominal pain (42.9%). The most common grade 3 or 4 adverse events were hypertension (14.3%), followed by proteinuria (9.5%), immune-mediated hepatitis (4.8%), and neutropenia (4.8). No treatment-related deaths occurred.

## 4. Discussion

To our best knowledge, this is the first real-world study to report the therapeutic efficacy and safety of TACE combined with atezo/bev for BCLC-B patients with HCC beyond the up-to-seven criteria. In the present study, the ORR was 61.9% per mRECIST and 42.9% per RECIST 1.1, whereas the median FPS was also not reached with a median follow-up of 11.7 months, and the safety profile was acceptable.

The high response rate found in our study may be caused by the synergistic effect of atezo/bev and TACE. For one thing, tumor cell death induced by TACE could release tumor antigens, inflammatory cytokines, and damage-associated molecular patterns that promote antigen presentation and initiate anti-tumor lymphocytes [13]. For another thing, although VEGF would increase after the treatment of TACE, which might inhibit anti-tumor immunity by limiting the function of T cells [22, 23]. Bevacizumab, as a VEGF inhibitor, could be against anti-tumor immunity induced by TACE and convert the immunosuppressive tumor microenvironment (TME) to an immunosupportive one [24].

Despite the observation period in our study was not long enough, TACE and atezo/bev could be a favorable therapeutic response. A literature-based meta-analysis showed that objective response measured by mRECIST could predict overall survival for patients receiving locoregional therapies and ORR could be considered as primary endpoints in phase II trials as recommended by European Association for the Study of the Liver guideline [20, 25]. Our study showed a promising and favorable result with a 61.9% ORR per mRECIST.

The median PFS was not reached with a median follow-up time of 11.7 months, which indicated that more than half of the patients in this study did not experience disease progression in nearly one year. The median PFS of the IMbrave 150 trail for BCLC-B patients was 12.6 months [26]. Hiraoka et al. reported the efficacy of 95 BCLC-B patients with HCC beyond up-to-seven criteria who received the treatment of Atezo/Bev [12]. The ORR of this study was 17.7% and 42.5% per RECIST1.1 and mRECIST at six weeks, respectively. Median PFS was 8.0 months with a median observation period of 6.0 months. The results of PFS were not satisfactory for patients receiving TACE alone, especially for patients beyond up-to-seven criteria. In the TACTICS trial, the PFS was 9 months in patients with tumors beyond the up-to-7 criteria from TACE alone group, which is much shorter than sorafenib-TACE group [27]. Similar results were reported in another study for patients received the treatment of lenvatinib combined with TACE compared with TACE alone [7]. These results indicated that locoregional therapies combined with systemic therapy might be optimal selection in patients with intermediate stage beyond up-to-7 HCC. TACE combined with other kinds of tyrosine kinase inhibitors (TKIs) and immune checkpoint inhibitors (ICIs) also present impressive efficacy with the treatment of unresectable HCC [17, 18, 28, 29]. Results from a multicenter retrospective study show that 13 BCLC-B and 39 BCLC-C patients received the treatment of TACE combined with lenvatinib plus sintilimab achieved an ORR of 46.7% per mRECIST with a median PFS and OS of 13.3 and 23.6 months. Meanwhile, several combination treatment regimens (including TACE, TKIs, and ICIs) for intermediate HCC are ongoing in phase III studies [14]. Our results suggest that TACE combined with atezo/bev may be a promising treatment regimen for intermediate HCC, especially for HCC beyond up-to-7 criteria.

Although the combination therapy regimen could improve tumor control, the optimal treatment order remains unclear. TACE first could lead to tumor necrosis and tumor antigen release, which might enhance the therapeutic effect of atezo/bev. On the other hand, performing atezo/bev prior to TACE may help to select responders to this treatment regimen considering that median time to response in IMbrave150 trail is 2.8 months [30]. In our study, we perform TACE first and only when liver function returns to Child-Pugh class A or B7, atezo/bev was initiated. This treatment order might help to enhance antitumor response through immunogenicity released by the procedure of TACE. Notably, the median TACE sessions were 2 (range 1–4) in this study, most of the patients (85.7%) received no more than two sessions of TACE to decrease the risk of worsening liver function. All these considerations might help to maximize the potential synergy of TACE and atezo/bev whilst limiting treatment-related toxicities.

The safety profile in our study is also investigated and consistent with previous reports [11, 31]. The most common adverse events were fever, AST elevation, ALT elevation, fatigue, and abdominal pain. The combination therapy might increase the rate of certain toxicities inevitably, but most cases were mild in severity. Hypertension was the most common adverse event associated with bevacizumab, 3 patients developed grade 3 or higher hypertension. Antihypertensive medicines were given without reduction or discontinuation. TACE combined with bevacizumab might increase the risk of gastrointestinal tract hemorrhage [32]. No similar results are found in our study, which may be due to lower frequency of TACE cycles as reported by Britten et al. [33, 34].

Although promising therapy responses were identified in the present cohort, the study has several limitations. The follow-up period was not long enough for evaluation of PFS and OS, and this is a retrospective study with small sample size. However, patients enrolled in this study are high homogeneity; all of the patients are classified as up-to-7 criteria. And this allows for the evaluation of the efficacy and safety of this combination treatment regimen.

In conclusion, TACE in combination with atezo/Bev indicated encouraging efficacy and an acceptable safety profile, making it a promising option for the treatment of BCLC-B patients with HCC beyond the up-to-seven criteria. This therapy regimen will be further studied in our larger prospective single-arm trial (ChiCTR2100049829).

## Figures and Tables

**Table 1:** Baseline characteristics of study patients.

Characteristics	All (*n* = 21)
Age (years), median (range)	56.0 (32–72)
Gender	
Female	2 (9.5)
Male	19 (90.5)
ECOG PS	
0/1	21 (100)
2	0 (0)
Child-Pugh score	
A	20 (95.2)
B7	1 (4.8)
mALBI grade	
1	10 (47.6)
2a	9 (42.9)
2b	2 (9.5)
Etiology	
HBV	18 (85.7)
Nonviral	3 (14.3)
Liver cirrhosis	
Yes	17 (81.0)
No	4 (19.0)
Varices	
Yes	16 (76.2)
No	5 (13.8)
AFP (ng/mL)	
<400	16 (76.2)
≥400	5 (13.8)
Tumor number	
2-3	4 (19.0)
≥3	17 (81.0)
Maximum tumor diameter (cm), median (range)	6.0 (1.3–18)
No. of TACE, median (range)	2 (1–4)
Cycle of atezo/bev, median (range)	9 (1–20)

**Table 2:** Summary of efficacy outcomes.

Variables, *n* (%)	All patients (*n* = 21)	
	Best overall response (mRECIST)	Best overall response (RECIST v1.1)
CR	6 (28.6)	0 (0)
PR	7 (33.3)	9 (42.9)
SD	8 (38.1)	12 (57.1)
PD	0 (0)	0 (0)
ORR	13 (61.9)	9 (42.9)
DCR	21 (100)	21 (100)
DRR ≥3 months	12 (57.1)	9 (42.9)
DRR ≥6 months	10 (47.6)	8 (38.1)

CR, complete response; PR, partial response; SD, stable disease; PD, progress disease; ORR, objective response rate; DCR, disease control rate; DRR, durable response rate.

**Table 3:** Summary of the treatment-related adverse events in patients (*n* = 21).

AE term, *n* (%)	Any grade	Grade 3/4
Any adverse event	21 (100)	7 (33.3)
Fever	15 (71.4)	0
Neutropenia	13 (61.9)	1 (4.8)
AST level increased	14 (66.7)	0
ALT level increased	13 (61.9)	0
Abdominal pain	9 (42.9)	0
Vomiting	5 (23.8)	0
Rash	4 (19.0)	0
Anemia	3 (14.3)	0
Hyperbilirubinemia	5 (23.8)	0
Hypertension	7 (33.3)	3 (14.3)
Fatigue	10 (47.6)	0
Proteinuria	4 (19.0)	2 (9.5)
Diarrhea	3 (14.3)	0
Pruritus	1 (4.8)	0
Oral ulcer	2 (9.5)	0
Immune-mediated hepatitis	2 (9.5)	1 (4.8)
Constipation	1 (4.8)	0
Thrombocytopenia	4 (19.0)	0
Decreased appetite	4 (19.0)	0
Hypothyroidism	3 (14.3)	0
Weight decreased	1 (4.8)	0
Abdominal distention	1 (4.8)	0
Oulorrhagia	1 (4.8)	0

AE, adverse event; AST, aspartate transaminase; ALT, alanine aminotransferase.

## Data Availability

The data that support the findings of this study are available from the corresponding author upon reasonable request.

## Abbreviations

HCC: Hepatocellular carcinoma
BCLC: Barcelona clinic liver cancer
TACE: Transarterial chemoembolization
atezo/bev: Atezolizumab plus bevacizumab
ORR: Objective response rate
RECIST: Response Evaluation Criteria in Solid Tumors
VEGF: Vascular endothelial growth factor
ECOG PS: Eastern cooperative oncology group performance status
AFP: Alpha-fetoprotein
PD: Progressive disease
OS: Overall survival
PFS: Progression-free survival
CR: Complete response
PR: Partial response
DRR: Durable response rate
CTCAE: Common terminology criteria for adverse events.
